# “Cellular agriculture”: current gaps between facts and claims regarding “cell-based meat”

**DOI:** 10.1093/af/vfac092

**Published:** 2023-04-15

**Authors:** Paul Wood, Lieven Thorrez, Jean-François Hocquette, Declan Troy, Mohammed Gagaoua

**Affiliations:** Monash University, Clayton, Victoria 3800, Australia; Tissue Engineering Lab, Department Development and Regeneration, KU Leuven Kulak, Kortrijk, Belgium; INRAE, University of Clermont Auvergne, Vetagro Sup, UMR Herbivores, Theix, 63122, Saint-Genès-Champanelle, France; Monash University, Clayton, Victoria 3800, Australia; PEGASE, INRAE, Institut Agro, 35590 Saint-Gilles, France

**Keywords:** animal and alternative proteins, “Cellular agriculture”, “Cultured meat”, food biotechnology, future foods, precision fermentation

**Implications**

- There has been a significant increase in the number of scientific articles related to “cell-based meat” (‘CBM’), which is in line with the current interest from the scientific community and consumers, but mainly from investors, food industry, and regulatory bodies.
- Despite the billions of dollars being invested in “cellular agriculture”, there are significant technical, ethical, regulatory, and commercial challenges to getting these products widely available in the market. In addition, the widespread adoption of such technologies can exacerbate global inequity between affluent and poor individuals and between high- and low-income countries.
- Current ‘CBM’ products are not identical to the products they aim to replace. First, there is still considerable dissimilarity at the level of sensory, nutritional, and textural properties, while important quality-generating steps in the conversion of muscle into conventional meat are missing. Second, many societal roles of animal production beyond nutrition can be lost, including ecosystem services, co-product benefits, and contributions to livelihoods and cultural meaning.
- Detailed production procedures are not available, making it impossible to corroborate the many claims related to their product characteristics and sustainability.
- ‘CBM’ companies arguing that the cost of all technology will eventually be significantly reduced often quote Moore’s law. However, biological systems like ‘CBM’ have natural limits and feedback mechanisms that negate this law.

## Introduction

In alignment with an emerging Silicon Valley–style outlook on the future of food, a bold 2019 report by the think tank RethinkX claimed that by 2030 the U.S. meat and dairy industries would be bankrupt due to “cellular agriculture” taking over their traditional markets (Tubb and Seba, 2019). This claim was based on their view of how quickly precision fermentation and “cell-based meat” (‘CBM’) technology would be developed and scaled, so they could compete on price parity with traditional livestock production (Leroy et al., 2023). However, estimates on future evolutions differ wildly. For example, a 2018 report ordered by the Flemish government predicted that the consumption of “clean meat” may start in approximately 2040 (van Diepen et al., 2018). Ten years ago, however, it was already touted that “clean meat” would be available in the market by 2017 (EC, 2012). By now, it is clear that it is difficult to make predictions and that many technical challenges remain before such products can become commercially available. Nevertheless, over the past few years, billions of dollars have been invested in these technologies related to cellular agriculture (including precision fermentation and CBM) and hundreds of new start-ups have been created around the globe (Boukid and Gagaoua, 2022). The terminology for developed products is still under discussion; for recognizability, we will use the term “cell-based meat”, though the term “meat” imparts characteristics that have not been proven, as we will discuss. The reasons for proposing new protein alternatives, including ‘CBM’, are diverse and divergent, but mainly related to ethical concerns about animal welfare and the possible impact of animal protein production on the environment (Siddiqui et al., 2022). This paper briefly describes the technical, regulatory, and consumer challenges facing both precision fermentation and CBM and examines their potential to disrupt the meat and dairy industries, with a focus on ‘CBM’ as an alternative to farm animal proteins.

## Precision Fermentation to Engineer Proteins for Dairy and Meat Industries

Precision fermentation is the process of engineering the gene sequence for a specific protein into a bacterium or yeast strain and then growing that strain in large-scale fermenters, to produce the required protein. This technology has been used for decades in the biotechnology sector. It was previously referred to as “recombinant protein production” and is used for many vaccines and drugs, such as insulin (Wood and Tavan, 2022).

In the food sector, precision fermentation has been used for decades to produce enzymes for cheese making or conventional fermentation. Chymosin, used in cheese manufacturing for milk coagulation, was originally extracted from the stomachs of calves before manufacturers switched to a recombinant form of this enzyme (expressed in a range of organisms). Recently, companies have used this technology to produce key proteins for the food industry. Impossible Foods uses a precision-fermentation form of hemoglobin to give their plant-based burgers the look and smell of red meat when they are cooked. As another example, The Every Company is producing chicken-free egg products using precision fermentation technology.

Until now, the major focus has been on dairy products. Around 60% of the companies in the precision fermentation space are focusing on the production of key dairy proteins. Perfect Day was the first company to release a commercial dairy product containing β-lactoglobulin. Other companies are now following their lead. One of the goals for companies like All G Foods and Eden Brew is to recreate a liquid milk, which contains both the whey and casein proteins that are needed to form a micelle, to give this product the full functionality of cow’s milk. These products will still need to have added fats, sugars, minerals, and vitamins to approach the nutritional content of cow’s milk.

There has been significant investment in the precision fermentation space and many predictions that this technology is going to disrupt the traditional meat and dairy industries; however, there are many technical, regulatory, and consumer challenges that need to be addressed. The major technical challenge will be the cost of goods, with precision fermentation being significantly more expensive. For milk proteins, a range of yeast strains can produce recombinant proteins at a rate of 10–30 g/l, but these proteins then need to be separated from the yeast cells and cell debris using a variety of downstream processing techniques that can account for up to 60% of the cost of manufacture. Precision fermentation technology will also be critical for the ‘CBM’ sector to produce the various growth factors and perhaps other compounds required to culture mammalian cells. To scale-up precision fermentation, companies use fermenters at >100,000-l capacity, which will require complex engineering and energy intensive processors.

In the USA, the regulatory process is relatively straightforward with the ability to use the ‘Generally Recognized As Safe’ classification. In Europe, however, it will be difficult to register precision fermentation products under the current legislative constellation, as they use genetically modified organisms in the manufacturing process. Finally, the labeling of precision fermentation products will vary considerably in different regions, and this has the potential to confuse consumers who are cautious of genetically modified products. Based on these issues, precision fermentation will be unlikely to disrupt the livestock industry but may provide high-value products for niche markets.

## How Close Does “Cell-Based Meat” Currently Come to Meat?

**Figure 1.**
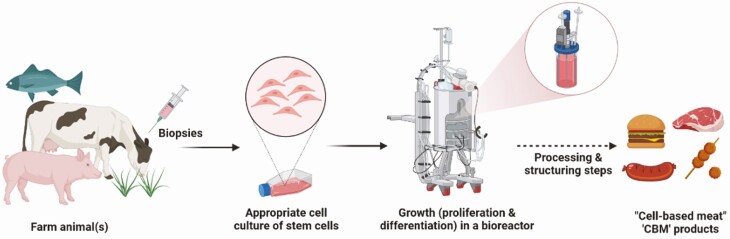
Simplified schematic representation of how ‘cell-based meat’ is touted to be produced using ‘cellular agriculture’ without rearing animals.

Companies aspire to produce meat without using animals (Figure 1). It is touted that such ‘CBM’ will be the same as meat from farm animal(s). Meat from animals is typically derived from the skeletal muscle of slaughtered animals, though other tissues such as liver and products of the fifth quarter are also consumed, of which the amounts depend on the region in the world and local food cultures. Here, we will focus on meat derived from skeletal muscle. Currently, many hurdles remain to make ‘CBM’ despite several decades of work stemming mainly from the fields of regenerative medicine and monoclonal antibody production. These hurdles have been summarized in Thorrez and Vandenburgh (2019) and still remain. The envisioned production procedures tend to oversimplify the complexity and growth of skeletal muscle. Skeletal muscle is a tissue which is composed of several cell types, the most abundant one being myofibers. Other cell types include connective tissue cells (fibroblasts), fat cells (adipocytes), endothelial cells, and blood cells. The current focus is mainly on the expansion of myoblasts, the precursor cells to myofibers. However, it is still unclear how long cells from biopsies can be expanded, as these primary cells undergo senescence during long-term expansion. Other precursor cell types (e.g., pluripotent cells) are being explored, but the creation of these cells, as well as the efficient differentiation towards myoblasts, currently involves genetic engineering.

Sometimes, the expansion phase is compared to fermentation. However, there is a stark difference between growth rates of bacteria and yeast vs. animal cells. Yeast can expand well over 1000× in less than a day, whereas this takes over 10 days for animal cells. Adding other cell types, in a way that spatially is similar to muscle, involves a co-culture setup, which adds to the complexity. Myoblasts need to fuse to form multi-nucleated myotubes and these myotubes are an intermediate towards myofibers, which occurs in animals during prenatal development. These myotubes are aligned (driven by unidirectional forces between attachments to the bones) and to create a similar alignment for ‘CBM’, manufacturing techniques and edible scaffold materials need to be developed. After birth, the myofibers then grow in volume and contractile strength since the organism is actively using them. The properties of muscle from fetuses and newborns (which seldomly is consumed) are vastly different from the muscle of adult animals. Building up proteins from the contractile apparatus, which are characteristics for skeletal muscle, requires the prolonged stimulation of muscle. Such stimulation is not currently accounted for and will require bioreactor development which will significantly increase the envisioned production time. As there is no product nor protocol available, most of the claims related to the production of ‘CBM’ in view of sustainability improvements (e.g., energy or water use) seem not scientifically substantiated or remain at best speculative, especially for its environmental footprint (Lynch and Pierrehumbert, 2019; Rodríguez Escobar et al., 2021).

### Manufacturing challenge for cell-based meat

The ‘CBM’ products are not commercialized because the current industrial production is still not economically viable due to the high production costs as well as lack of regulatory framework. In fact, one of the greatest challenges facing the scaling of ‘CBM’ manufacturing is the cost of goods for these products. This is driven primarily by the cost of the culture media, the need for high quality facilities, and the capital cost for sophisticated manufacturing facilities. The biotechnology industry has been using cell-based systems for the manufacture of monoclonal antibody therapeutics for several decades and this is an expensive technology. The use of serum-free media is standard but more expensive than the use of serum, due to the cost of the recombinant growth factors.

With ‘CBM’, the final product of cell culture will be the cells themselves, which can be used as a cell slurry or induced to undergo muscle fibers differentiation. Once this step has occurred, the cells will be exceedingly difficult to handle, and this part of the manufacturing process is yet to be fully described. Many ‘CBM’ companies are claiming that the costs will be dramatically reduced using large-scale bioreactors at up to a 250,000-l scale. However, the only product registered so far is produced at a 5-l scale, so validating these systems at scale will be a major challenge. In addition, the use of antibiotics will not be permitted, and these larger scale fermentations will require around 90-days continuous sterile culture. Another claim is that ‘CBM’ manufacturing will not require the level of biosecurity used in the biopharma industry, yet most of these quality requirements are driven by the need to maintain sterile systems.

The cost of large-scale ‘CBM’ facilities has been estimated at $600M U.S. and the depreciation costs of these facilities will be a major component of the final cost of goods. While interesting, other technology that is being developed for ‘CBM,’ like edible cell-scaffolds and 3D printing with multiple cell types, just add to the challenge of scaling and cost of goods. The development of blended products with plant-based material will help to reduce costs and stabilize formulation. Some companies are focusing on culturing fat cells, with the view that they will only need to add 5% of these cells to their formulations to give the product the hint of meat.

Proponents of CBM frequently use the concept of Moore’s Law, the idea that the cost of production for new technologies decreases exponentially over time, to argue that this will also be the case with CBM. However, while Moore’s law has been predictive with the cost of production for physical technologies like computers and high-throughput omics methods, it has never been applied to a biological system due to the complexity of the biological events and mechanisms underpinning cell growth.

### Nutritional challenges for cell-based products

**Figure 2.**
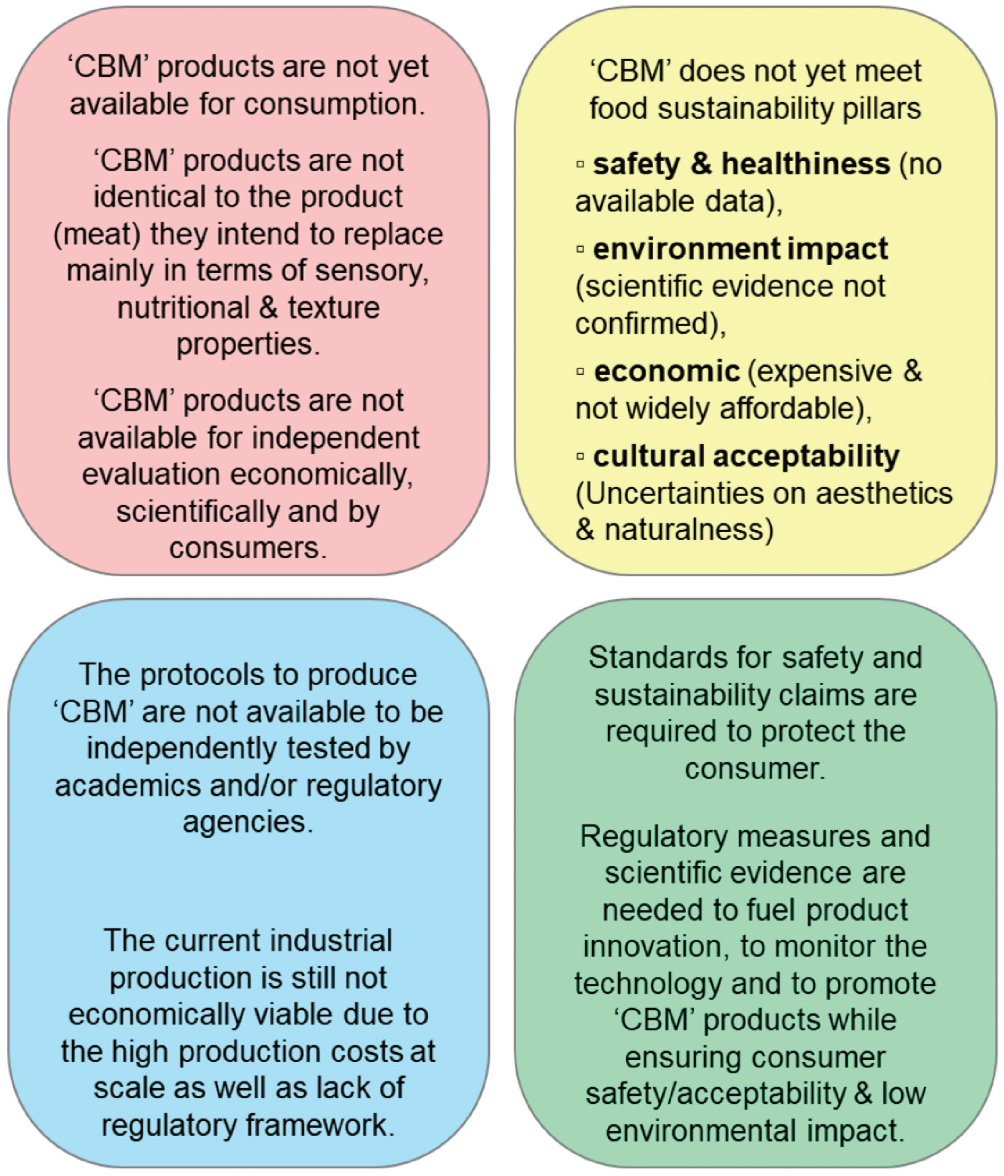
Summary of the remaining challenges and questions related to the future of ‘cell-based meat’ (‘CBM’).

At present, despite the claims of companies, such products are not ready for the market. To the best of our knowledge, only one lab-based food product is registered (in Singapore) and has been temporarily available in very limited quantities. In addition, production protocols are not available for independent testing by academics or regulatory agencies (Figure 2). Therefore, any claims related to nutritional content cannot be verified as such. Indeed, much remains to be inferred based on available research-scale protocols, as was conceptually analyzed by Fraeye et al. (2020). Sensory properties of meat such as texture, color, and flavor can perhaps be adjusted with food engineering techniques to create products with similar appearance to meat. However, the use of these products in downstream cooking applications may be limited as these characteristics may change during further processing steps such as heating and interaction with other ingredients. It is much harder to make statements related to nutritional characteristics, although these products can be suitable for people having adverse reactions to peas, soy, and gluten. Meat contains highly digestible proteins with essential amino acids, vitamins, and minerals (see elsewhere in this Special Issue). However, while nutrients can be added to meat replacement products, the cost (both in economic and sustainability terms) of this is unknown. Moreover, simply adding components may lead to a different bioavailability, for example, the extent and rate by which they are absorbed by the body.

## Regulatory and Consumer Issues Related to “Cell-Based Meat” Consumption

As described by Chriki et al. (2022), an important question pertains to the legal nature of ‘CBM’: is it really meat? According to the American Meat Science Association (Boler and Woerner, 2017), the European regulation (Annex I of Regulation No. 853/2004), and other legal definitions (Ong et al., 2020), meat comes from a part (muscles and/or edible tissues) of an animal consumed as food. Therefore, ‘CBM’ does not currently qualify as meat except if we consider living cells as part of an animal (Ong et al., 2020). To be considered meat, ‘CBM’ must be sourced from an animal, proven to be safe for consumption, and be similar in composition, nutritional value, and sensory quality to meat from farmed animals, which is not yet the case (Boler and Woerner, 2017). In addition, to consider ‘CBM’ as a *novel food* (within EU legislation) means that it should be safe and properly labeled, so as not to mislead consumers.

From a biological point of view, meat is the final product of aged muscle through a maturation process (a well-known process by butchers), just as wine originates from grape juice through winemaking. The current cellular agriculture process, however, produces muscle fibers/cells and not meat. Therefore, consumers who are familiar with what meat represents within their culinary and agricultural legacies tend to decline calling it “meat”, unlike vegan activists who see it as a welcome strategy to eliminate meat from the food system and, therefore, wish to give it maximum market potential by capturing the meat category (Gousset et al., 2022).

Additionally, various societal roles of animal production beyond nutrition can be lost in the process. This includes the many ecosystem services offered by livestock, the generation of edible and non-edible co-products (e.g., hides, wool, manure, draught power, serum, blood, and fats), contributions to livelihoods, and the generation of cultural meaning. Finally, still from a societal perspective, it has been argued that ‘CBM’ may exacerbate global inequity, including increased economic disparity between regions (Mahoney, 2022). The technology risks being under the control of multinational corporations (based on patents and high technological, economic, and legal entry barriers), may lead to the creation of luxury foods, and cause a further expansion of food deserts (Mahoney, 2022).

When conducting surveys in view of market acceptability, answers are often inconsistent (Gousset et al., 2022). This is not entirely unexpected, as consumers are being asked about a product which does not yet exist, with the exception of Singapore. In addition, most may not know what ‘CBM’ is and confuse it with any other type of artificial meat (e.g., plant-based imitations). Willingness to “try the product” and willingness to “consume it regularly” are also often confounded. Indeed, many respondents would like to taste the product once for curiosity, which does not mean they would consume it regularly for varied reasons. Therefore, the acceptability of ‘CBM’ is often overestimated because it is based on willingness to try, and not on intentions to eat ‘CBM’ regularly. Many surveys confirm that respondents who express a high acceptance tend to be young, urban, and highly educated at least in some major countries but not all, possess little factual knowledge about ‘CBM’ production, and are already inclined to reduce meat consumption (for a variety of reasons including concerns about animal welfare and environmental issues). Conversely, older, and less urban consumers are more reluctant and express concerns about the future of the countryside, livestock farming, and landscape and pastures. Such consumers also highlight the unnaturalness and low healthiness of the product and express a higher emotional resistance. In any case, willingness to pay is low overall, since most respondents (68% in France, 71% in Brazil, and 86% in China) were willing to pay less for ‘CBM’ compared to conventional meat (Ellies-Oury et al., 2022). All these motives and barriers may differ from country to country with barriers being stronger in those regions holding stronger traditional values and motives being stronger where the challenges related to food demand are the greatest, such as in Asia and Africa.

## Conclusions

“Cellular agriculture”, including ‘CBM’ and precision fermentation, has been promoted as an alternative for producing future food proteins by replacing dairy and meat without involving animals. The development of such novel technologies, despite several ethical concerns, is accompanied by a multitude of research reports, the creation of start-ups, massive investments, and prominent media coverage. It has evolved into a hot topic for societal debates, often associated with divergent opinions. Currently, however, these new food products are not available for consumption in meaningful amounts, nor are they exposed to independent evaluation on economic or scientific grounds. Indeed, the multitude of technical challenges related to the scalability and production of ‘CBM’ prototypes are not available for accurate and independent assessment in terms of their sustainability, intrinsic (sensory, nutritional, and technological attributes) or extrinsic quality. The perception of unnaturalness and the low or poor cultural acceptance by consumers, mainly because of lack of familiarity and uncertainties about the aesthetics, are other barriers to societal acceptance. Therefore, they are well perceived only by a certain category of people (niche market or animal activist groups). The other drawbacks of these products are the limited data on the long-term human health implications (safety and health), environmental impact (although they claim less land usage), and obscure risks related with cellular engineering. Streamlined regulatory measures and continued basic research, free of conflicts of interest, are necessary to fuel product innovation, set forth requirements for appropriate monitoring of these innovative technologies, and to promote such novel foods while simultaneously ensuring both consumer safety/acceptability and guaranteeing low environmental impact.

Finally, we believe that the need for more proteins to feed the world’s growing population will continue to be the main driver of innovation in the production of proteins and meat alternatives. We must keep in mind, however, that most of the growth in population will be in developing countries. Thus, major challenges that need to be further considered in the development of such novel foods from “cellular agriculture” origin will be their price and distribution logistics as well as how they are situated in a fair and affordable food equity framework.

## About the Author(s)

**Paul Wood** has led R&D teams from CSIRO, CSL, and Pfizer Animal Health, and was Deputy-Director of the Vaccine Technology CRC. In recognition for his innovative work, he received the CSIRO Medal and Clunies Ross award and was made an Officer in the Order of Australia. He is Chair of the Global Alliance for Livestock Veterinary Medicines and an Insect farming start-up, part of the Boards of Dairy Australia and the Australian Academy Technology, Science and Engineering, Adjunct Professor at Monash University, and a mentor for various AgTech accelerators (e.g., Sprout X, Rocket Seeder, CSIRO Protein Mission).

**Lieven Thorrez** has a multidisciplinary background in biomedical sciences, representing over two decades of experience with animal cell cultures, including the development of viral vectors for targeted gene delivery, stem cell biology, muscle tissue engineering, applied bioinformatics, systems biology, and evolutionary biology. His current research deals with cell isolation, expansion, and characterization, the engineering of a blood vessel network in muscle, methods for muscle stimulation, and de- and recellularization methods towards applications (drug testing, regenerative medicine, and cultured meat production). His laboratory engages in national and European projects and collaborates with industry, offering tools for drug testing and consulting services.

**Jean-François Hocquette** is a scientist at INRAE, France, and President of the French Association for Animal Production. His research interests concern muscle biology, genomics, and consumer studies. He was leader of the “Muscle Growth and Metabolism” research team (1999–2006) and Director of the Herbivore Research Unit (172 staff, 2006–2010). He is an active member of the European Federation of Animal Science (EAAP) and editor of the French Meat R&D journal. He was awarded by the French Meat Academy (2010) and twice by the American Society of Animal Science (2014 and 2021).

**Declan Troy** is Assistant Director of Research at Teagasc, Ireland, where he also is Director of Technology and Knowledge Transfer for its Food Programme and Head of the Ashtown Food Research Centre. His research focuses on the biochemistry and role of muscle proteins in meat tenderness. As President of the Institute of Food Science and Technology of Ireland, he was Chairman of the World Congress of Food Science and Technology 2016 in Dublin. He has also been appointed (2019) as head of the International Secretariat of the International Congress of Meat Science and Technology and is Academic Leader of Meat Technology Ireland.

**Mohammed Gagaoua** is a meat (animal/food) scientist at PEGASE research unit, INRAE, France. He develops research activities on meat quality and production systems, as well as on muscle biology and biochemistry of meat in relation to its eating qualities, up to consumer level. He has expertise in foodomics (MeatOmics) and statistical approaches that consider the main factors affecting meat quality including production systems and post-slaughter harvesting practices. Mohammed has respected knowledge in data integration in the context of the farm-to-fork continuum. He was runner-up in 2015 for the International Meat Secretariat Award. In 2018, he received the award for outstanding contributions to advancing meat science at the International Congress of Meat Science and Technology (ICoMST) as well as the Meat Science journal award for best presentation.
